# Aqua­chlorido(2,2′:6′,2′′-terpyrid­yl)copper(II) chloride monohydrate

**DOI:** 10.1107/S160053681003391X

**Published:** 2010-08-28

**Authors:** Laurette Schmitt, Gaël Labat, Helen Stoeckli-Evans

**Affiliations:** a Institute of Physics, University of Neuchâtel, rue Emile-Argand 11, CH-2009 Neuchâtel, Switzerland

## Abstract

The title complex, [CuCl(C_15_H_11_N_3_)(H_2_O)]Cl·H_2_O, is composed of a monocation that possesses mirror symmetry. The Cu^II^ atom has a distorted square-pyramidal geometry, being coordinated by the three N atoms of the terpyridine ligand and a Cl atom in the equatorial plane, and by a water mol­ecule O atom in the axial position. The charges are balanced by a chloride anion positionally disorded over two positions related by the mirror symmetry. The compound crystallizes as a monohydrate, with the water mol­ecule also being positionally disordered over two positions related by the mirror symmetry. In the crystal, the various components of the complex are linked *via* O—H⋯O and O—H⋯Cl hydrogen bonds, forming a two-dimensional network in the *ab* plane. There are also a number of C—H⋯Cl and C—H⋯O inter­actions which stabilize the crystal structure.

## Related literature

For details of the Cambridge Structural Database, see: Allen (2002 ▶). For the structure of a related compound, see: Koo *et al.* (2003 ▶). For the τ descriptor for 5-coordination, see: Addison *et al.* (1984 ▶); Spek (2009 ▶).
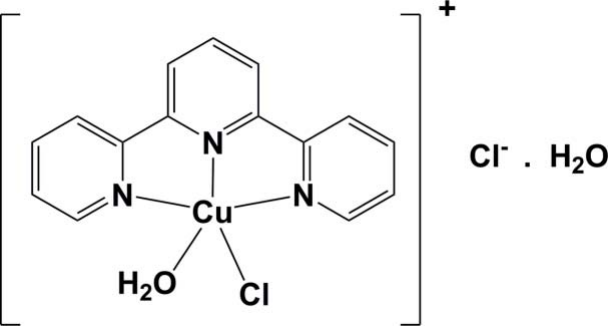


## Experimental

### 

#### Crystal data

[CuCl(C_15_H_11_N_3_)(H_2_O)]Cl·H_2_O
*M* *_r_* = 403.74
Monoclinic, 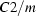

*a* = 9.7155 (8) Å
*b* = 13.6929 (8) Å
*c* = 12.6599 (10) Å
β = 107.532 (6)°
*V* = 1606.0 (2) Å^3^
*Z* = 4
Mo *K*α radiation
μ = 1.70 mm^−1^
*T* = 173 K
0.40 × 0.40 × 0.10 mm

#### Data collection

Stoe IPDS 2 diffractometer
Absorption correction: multi-scan (*MULscanABS* in *PLATON*; Spek, 2009 ▶) *T*_min_ = 0.688, *T*_max_ = 1.000
14960 measured reflections
2267 independent reflections
2027 reflections with *I* > 2σ(*I*)
*R*_int_ = 0.033

#### Refinement

*R*[*F* ^2^ > 2σ(*F* ^2^)] = 0.030
*wR*(*F* ^2^) = 0.079
*S* = 1.09
2267 reflections
131 parameters
4 restraints
H atoms treated by a mixture of independent and constrained refinement
Δρ_max_ = 0.59 e Å^−3^
Δρ_min_ = −0.70 e Å^−3^

### 

Data collection: *X-AREA* (Stoe & Cie, 2006 ▶); cell refinement: *X-AREA*; data reduction: *X-RED32* (Stoe & Cie, 2006 ▶); program(s) used to solve structure: *SHELXS97* (Sheldrick, 2008 ▶); program(s) used to refine structure: *SHELXL97* (Sheldrick, 2008 ▶); molecular graphics: *PLATON* (Spek, 2009 ▶) and *Mercury* (Macrae *et al.*, 2006); software used to prepare material for publication: *SHELXL97*.

## Supplementary Material

Crystal structure: contains datablocks I, global. DOI: 10.1107/S160053681003391X/om2357sup1.cif

Structure factors: contains datablocks I. DOI: 10.1107/S160053681003391X/om2357Isup2.hkl

Additional supplementary materials:  crystallographic information; 3D view; checkCIF report

## Figures and Tables

**Table 1:** Hydrogen-bond geometry (Å, °)

*D*—H⋯*A*	*D*—H	H⋯*A*	*D*⋯*A*	*D*—H⋯*A*
O1*W*—H1⋯Cl2^i^	0.80 (2)	2.34 (2)	3.143 (2)	175 (2)
O1*W*—H1⋯O2*W*^i^	0.80 (2)	1.99 (2)	2.787 (8)	170 (2)
O2*W*—H2*A*⋯O2*W*^ii^	0.84 (2)	2.13 (3)	2.922 (15)	159 (6)
C2—H2⋯Cl2^iii^	0.95	2.69	3.635 (2)	172
C5—H5⋯Cl2^iv^	0.95	2.65	3.593 (3)	172
C5—H5⋯O2*W*^iv^	0.95	2.50	3.429 (8)	166
C7—H7⋯Cl1^v^	0.95	2.82	3.765 (2)	175

Symmetry codes: (i) 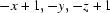; (ii) 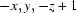; (iii) 
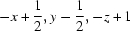
; (iv) 
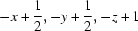
; (v) 
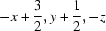
.

**Fig. 1.**
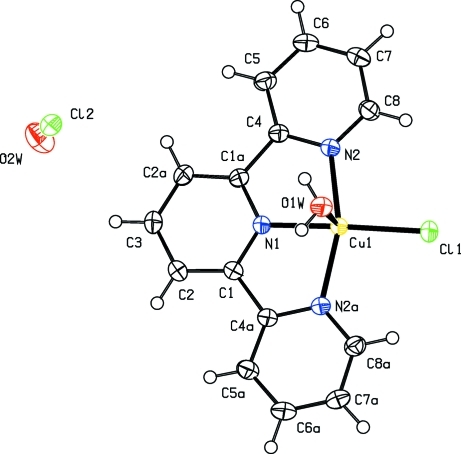
A view of the molecular structure of compound (I) with the displacement ellipsoids drawn at the 50% probability level [Symmetry code: (a) = x, -y, z; the H-atoms of the disordered water molecule of crystallization (O2w), and the symmetry related Cl- anion (Cl2a) and water molecule (O2wa) are not shown].

**Fig. 2.**
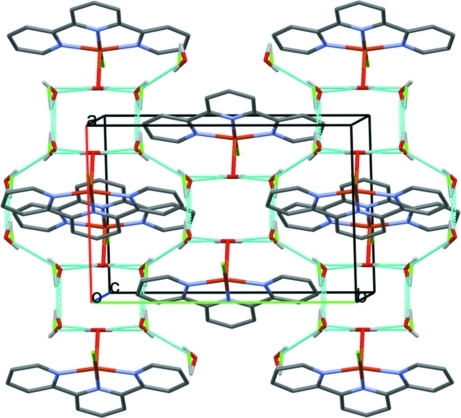
A view along the c axis of the crystal packing of compound (I). The O—H···O and O—H···Cl hydrogen bonds are shown as dashed cyan lines (see Table 1 for details; H-atoms not involved in hydrogen bonding have been omitted for clarity).

## supplementary crystallographic information

### Comment

The title compound, (I), was prepared as a by-product of the reaction of 2,2':6',2''-terpyridine (= terpy) with CuCl_2_ in the presence of sodium sulphite. A search of the Cambridge Structural Database (CSD, Version 5.1, last update May 2010; Allen *et al.*, 2002) for copper(II) terpyridine complexes with a water molecule coordinated to the copper(II) atom revealed 22 hits. With a chloride atom coordinated to the copper(II) atom 33 hits were obtained. Surprisingly, only one compound, involving bisterpy (= 2,2':4',4'':2'',2'''-quarterpyridyl, 6',6''-di-2-pyridine) was located with both a chloride and a water molecule coordinated to the copper(II) atom, namely [Cu_2_(bisterpy)(H_2_O)_2_Cl_2_]Cl_2_ (II) [Koo *et al.*, 2003].

The structure of compound (I) is illustrated in Fig. 1. It is composed of a [(H_2_OClCu(terp)]^+^ cation that possesses mirror symmetry (with atoms Cu1, Cl1, N1, O1W and C3 lying in the mirror plane), and a Cl^-^ anion. This anion, atom Cl2, is positionally disordered over two postions related by the mirror symmetry. A water molecule of crystallization, O2W, is also present and it too is positionally disordered over two postions related by the mirror symmetry. The bond distances and angles are similar to those in compound (II). For example, the Cu1—Cl1 and Cu1—O1W distances are 2.2255 (6) and 2.3372(19 Å, respectively, compared to 2.233 and 2.330 Å, respectively, in (II). The copper coordination sphere is distorted square pyramidal with a τ value of 0.17, compared to 0.18 in (II) [idealized values are 0 for square pyramidal and 1 for trigonal bipyramidal; Addison *et al.*, 1984; Spek, 2009].

In the crystal of (I) the cations are linked to the anions and the water molecules of crystallization by O—H···O and O—H···Cl hydrogen bonds resulting in the formation of a two-dimensional network (Table 1 and Fig. 2). In the crystal C—H···O and C—H···Cl interactions are also present (Table 1).

### Experimental

An aqueous solution (20 ml) of copper(II)chloride dihydrate (0.429 mmol, 75 mg) and 2, 2':6' 2''-terpyridine (0.429 mmol, 100 mg) was heated at 353 K for 1 h. After hot filtration the green solution was cooled to RT and sodium sulfite (1.717 mmol, 216 mg) was added. The resulting solution was left in the fridge for two months and green block-like crystals were obtained together with a small quantity of greenish-blue crystals. The latter were shown by X-ray diffraction analysis to be the title compound (I).

### Refinement

The chlorine anion (Cl2) and the water molecule of crystallization (O2W) were found to be split over two positions related by the mirror plane; they were refined with occupancies of 0.5 each. The water molecule H-atoms were located in a difference electron-density map and were refined with distance restraints of 0.84 (2) Å and *U*_iso_(H) = 1.5*U*_eq_(O). The C-bound H-atoms were included in calculated positions and treated as riding atoms: C—H = 0.95 Å with *U*_iso_(H) = 1.2*U*_eq_(C).

### Crystal data

**Table:**

[CuCl(C_15_H_11_N_3_)(H_2_O)]Cl·H_2_O	*F*(000) = 820
*M**_r_* = 403.74	*D*_x_ = 1.670 Mg m^−^^3^
Monoclinic, *C*2/*m*	Mo *K*α radiation, λ = 0.71073 Å
Hall symbol: -C 2y	Cell parameters from 17467 reflections
*a* = 9.7155 (8) Å	θ = 1.7–29.6°
*b* = 13.6929 (8) Å	µ = 1.70 mm^−^^1^
*c* = 12.6599 (10) Å	*T* = 173 K
β = 107.532 (6)°	Plate, blue-green
*V* = 1606.0 (2) Å^3^	0.40 × 0.40 × 0.10 mm
*Z* = 4	

### Data collection

**Table:**

Stoe IPDS-2 diffractometer	2267 independent reflections
Radiation source: fine-focus sealed tube	2027 reflections with *I* > 2σ(*I*)
graphite	*R*_int_ = 0.033
phi + ω scans	θ_max_ = 29.2°, θ_min_ = 1.7°
Absorption correction: multi-scan (MULscanABS in *PLATON*; Spek, 2009)	*h* = −13→13
*T*_min_ = 0.688, *T*_max_ = 1.000	*k* = −17→18
14960 measured reflections	*l* = −17→17

### Refinement

**Table:**

Refinement on *F*^2^	Secondary atom site location: difference Fourier map
Least-squares matrix: full	Hydrogen site location: inferred from neighbouring sites
*R*[*F*^2^ > 2σ(*F*^2^)] = 0.030	H atoms treated by a mixture of independent and constrained refinement
*wR*(*F*^2^) = 0.079	*w* = 1/[σ^2^(*F*_o_^2^) + (0.0448*P*)^2^ + 1.3613*P*] where *P* = (*F*_o_^2^ + 2*F*_c_^2^)/3
*S* = 1.09	(Δ/σ)_max_ < 0.001
2267 reflections	Δρ_max_ = 0.59 e Å^−^^3^
131 parameters	Δρ_min_ = −0.70 e Å^−^^3^
4 restraints	Extinction correction: *SHELXL97* (Sheldrick, 2008), Fc^*^=kFc[1+0.001xFc^2^λ^3^/sin(2θ)]^-1/4^
Primary atom site location: structure-invariant direct methods	Extinction coefficient: 0.0027 (7)

### Special details

**Table:**

Geometry. Bond distances, angles *etc*. have been calculated using the rounded fractional coordinates. All su's are estimated from the variances of the (full) variance-covariance matrix. The cell e.s.d.'s are taken into account in the estimation of distances, angles and torsion angles
Refinement. Refinement of *F*^2^ against ALL reflections. The weighted *R*-factor *wR* and goodness of fit *S* are based on *F*^2^, conventional *R*-factors *R* are based on *F*, with *F* set to zero for negative *F*^2^. The threshold expression of *F*^2^ > σ(*F*^2^) is used only for calculating *R*-factors(gt) *etc*. and is not relevant to the choice of reflections for refinement. *R*-factors based on *F*^2^ are statistically about twice as large as those based on *F*, and *R*- factors based on ALL data will be even larger.

### Fractional atomic coordinates and isotropic or equivalent isotropic displacement parameters (Å^2^)

**Table:**

	*x*	*y*	*z*	*U*_iso_*/*U*_eq_	Occ. (<1)
Cu1	0.62103 (3)	0.00000	0.15295 (2)	0.0228 (1)	
Cl1	0.71809 (7)	0.00000	0.01460 (5)	0.0313 (2)	
O1W	0.8290 (2)	0.00000	0.30520 (15)	0.0297 (5)	
N1	0.5024 (2)	0.00000	0.25225 (16)	0.0220 (5)	
N2	0.59110 (15)	0.14574 (10)	0.16522 (11)	0.0232 (3)	
C1	0.46408 (17)	−0.08528 (12)	0.28568 (13)	0.0236 (4)	
C2	0.38165 (18)	−0.08804 (13)	0.35839 (14)	0.0285 (5)	
C3	0.3403 (3)	0.00000	0.3937 (2)	0.0307 (7)	
C4	0.51479 (17)	0.16973 (12)	0.23508 (13)	0.0232 (4)	
C5	0.48556 (19)	0.26583 (13)	0.25435 (15)	0.0285 (5)	
C6	0.5360 (2)	0.33929 (13)	0.20053 (16)	0.0324 (5)	
C7	0.6149 (2)	0.31520 (13)	0.12996 (16)	0.0316 (5)	
C8	0.64036 (19)	0.21726 (13)	0.11436 (14)	0.0281 (5)	
Cl2	0.1811 (2)	0.17457 (12)	0.53617 (15)	0.0315 (4)	0.500
O2W	0.1572 (8)	0.1517 (5)	0.5452 (7)	0.0527 (19)	0.500
H1	0.826 (3)	−0.0471 (14)	0.3423 (18)	0.0450*	
H2	0.35450	−0.14850	0.38310	0.0340*	
H3	0.28300	0.00000	0.44270	0.0370*	
H5	0.43180	0.28120	0.30370	0.0340*	
H6	0.51640	0.40580	0.21210	0.0390*	
H7	0.65110	0.36470	0.09280	0.0380*	
H8	0.69460	0.20050	0.06590	0.0340*	
H2A	0.070 (2)	0.168 (4)	0.520 (6)	0.0790*	0.500
H2B	0.215 (5)	0.193 (4)	0.534 (7)	0.0790*	0.500

### Atomic displacement parameters (Å^2^)

**Table:**

	*U*^11^	*U*^22^	*U*^33^	*U*^12^	*U*^13^	*U*^23^
Cu1	0.0288 (2)	0.0179 (2)	0.0255 (2)	0.0000	0.0142 (1)	0.0000
Cl1	0.0424 (3)	0.0279 (3)	0.0310 (3)	0.0000	0.0223 (2)	0.0000
O1W	0.0352 (9)	0.0237 (8)	0.0312 (9)	0.0000	0.0114 (7)	0.0000
N1	0.0247 (9)	0.0203 (9)	0.0230 (8)	0.0000	0.0104 (7)	0.0000
N2	0.0261 (6)	0.0193 (6)	0.0255 (6)	−0.0001 (5)	0.0096 (5)	0.0007 (5)
C1	0.0253 (7)	0.0211 (8)	0.0252 (7)	−0.0019 (6)	0.0088 (6)	0.0004 (6)
C2	0.0327 (8)	0.0262 (8)	0.0302 (8)	−0.0034 (6)	0.0148 (7)	0.0022 (6)
C3	0.0351 (12)	0.0320 (13)	0.0311 (11)	0.0000	0.0191 (10)	0.0000
C4	0.0242 (7)	0.0201 (7)	0.0252 (7)	0.0008 (6)	0.0071 (6)	−0.0001 (6)
C5	0.0302 (8)	0.0226 (8)	0.0331 (8)	0.0015 (6)	0.0101 (7)	−0.0025 (6)
C6	0.0367 (9)	0.0195 (8)	0.0398 (9)	0.0003 (7)	0.0097 (7)	−0.0007 (7)
C7	0.0358 (9)	0.0226 (8)	0.0361 (9)	−0.0040 (7)	0.0103 (7)	0.0043 (7)
C8	0.0292 (8)	0.0251 (8)	0.0309 (8)	−0.0022 (7)	0.0106 (6)	0.0023 (6)
Cl2	0.0346 (7)	0.0289 (7)	0.0337 (5)	0.0011 (5)	0.0146 (5)	−0.0028 (5)
O2W	0.042 (3)	0.053 (4)	0.065 (3)	−0.001 (2)	0.019 (2)	0.015 (3)

### Geometric parameters (Å, °)

**Table:**

Cu1—Cl1	2.2253 (7)	N2—C8	1.337 (2)
Cu1—Cl1^i^	3.3383 (8)	N2—C4	1.355 (2)
Cu1—O1W	2.3348 (19)	C1—C4^ii^	1.477 (2)
Cu1—N1	1.945 (2)	C1—C2	1.391 (2)
Cu1—N2	2.0294 (14)	C2—C3	1.387 (2)
Cu1—N2^ii^	2.0294 (14)	C4—C5	1.383 (2)
Cl2—O2W	0.426 (8)	C5—C6	1.385 (3)
Cl2—H2B	0.42 (6)	C6—C7	1.381 (3)
Cl2—H2A	1.04 (3)	C7—C8	1.389 (3)
O1W—H1^ii^	0.80 (2)	C2—H2	0.9500
O1W—H1	0.80 (2)	C3—H3	0.9500
O2W—H2B	0.84 (6)	C5—H5	0.9500
O2W—H2A	0.84 (4)	C6—H6	0.9500
N1—C1	1.3328 (19)	C7—H7	0.9500
N1—C1^ii^	1.3328 (19)	C8—H8	0.9500
			
Cl1—Cu1—O1W	100.56 (5)	C2—C1—C4^ii^	126.85 (15)
Cl1—Cu1—N1	169.42 (6)	N1—C1—C2	120.37 (16)
Cl1—Cu1—N2	99.48 (4)	C1—C2—C3	118.05 (17)
Cl1—Cu1—N2^ii^	99.48 (4)	C2—C3—C2^ii^	120.8 (2)
O1W—Cu1—N1	90.02 (8)	N2—C4—C1^ii^	114.40 (14)
O1W—Cu1—N2	92.59 (4)	N2—C4—C5	121.85 (15)
O1W—Cu1—N2^ii^	92.59 (4)	C1^ii^—C4—C5	123.74 (16)
N1—Cu1—N2	79.87 (4)	C4—C5—C6	118.84 (17)
N1—Cu1—N2^ii^	79.87 (4)	C5—C6—C7	119.51 (17)
N2—Cu1—N2^ii^	159.07 (6)	C6—C7—C8	118.69 (17)
O2W—Cl2—H2B	163 (10)	N2—C8—C7	122.27 (17)
H2A—Cl2—H2B	145 (8)	C3—C2—H2	121.00
Cu1—O1W—H1^ii^	107.9 (19)	C1—C2—H2	121.00
H1—O1W—H1^ii^	107 (2)	C2—C3—H3	120.00
Cu1—O1W—H1	107.9 (19)	C2^ii^—C3—H3	120.00
H2A—O2W—H2B	114 (6)	C4—C5—H5	121.00
Cu1—N1—C1^ii^	118.81 (10)	C6—C5—H5	121.00
Cu1—N1—C1	118.81 (10)	C7—C6—H6	120.00
C1—N1—C1^ii^	122.37 (18)	C5—C6—H6	120.00
C4—N2—C8	118.84 (15)	C6—C7—H7	121.00
Cu1—N2—C8	127.08 (12)	C8—C7—H7	121.00
Cu1—N2—C4	114.06 (11)	C7—C8—H8	119.00
N1—C1—C4^ii^	112.75 (15)	N2—C8—H8	119.00
			
O1W—Cu1—N1—C1	−89.53 (15)	Cu1—N2—C4—C5	179.30 (13)
N2—Cu1—N1—C1	177.85 (17)	Cu1—N2—C4—C1^ii^	−1.98 (18)
N2—Cu1—N1—C1^ii^	−3.10 (15)	C8—N2—C4—C5	0.6 (2)
N2^ii^—Cu1—N1—C1	3.10 (15)	C8—N2—C4—C1^ii^	179.29 (15)
Cl1—Cu1—N2—C4	171.96 (11)	Cu1—N2—C8—C7	−179.12 (14)
Cl1—Cu1—N2—C8	−9.43 (15)	C4—N2—C8—C7	−0.6 (3)
O1W—Cu1—N2—C4	−86.88 (12)	N1—C1—C2—C3	0.5 (3)
O1W—Cu1—N2—C8	91.73 (15)	C4^ii^—C1—C2—C3	−177.64 (19)
N1—Cu1—N2—C4	2.68 (12)	C2—C1—C4^ii^—N2^ii^	178.71 (16)
N1—Cu1—N2—C8	−178.71 (16)	C2—C1—C4^ii^—C5^ii^	0.0 (3)
N2^ii^—Cu1—N2—C4	17.3 (2)	C1—C2—C3—C2^ii^	−0.8 (3)
N2^ii^—Cu1—N2—C8	−164.12 (15)	N2—C4—C5—C6	0.0 (3)
Cu1—N1—C1—C2	178.75 (13)	C1^ii^—C4—C5—C6	−178.62 (17)
Cu1—N1—C1—C4^ii^	−2.9 (2)	C4—C5—C6—C7	−0.5 (3)
C1^ii^—N1—C1—C2	−0.3 (3)	C5—C6—C7—C8	0.5 (3)
Cu1—N1—C1^ii^—C4	2.9 (2)	C6—C7—C8—N2	0.0 (3)
C1—N1—C1^ii^—C4	−178.13 (17)		

Symmetry codes: (i) −*x*+1, *y*, −*z*; (ii) *x*, −*y*, *z*.

### Hydrogen-bond geometry (Å, °)

**Table:**

*D*—H···*A*	*D*—H	H···*A*	*D*···*A*	*D*—H···*A*
O1W—H1···Cl2^iii^	0.80 (2)	2.34 (2)	3.143 (2)	175 (2)
O1W—H1···O2W^iii^	0.80 (2)	1.99 (2)	2.787 (8)	170 (2)
O2W—H2A···O2W^iv^	0.84 (2)	2.13 (3)	2.922 (15)	159 (6)
C2—H2···Cl2^v^	0.95	2.69	3.635 (2)	172
C5—H5···Cl2^vi^	0.95	2.65	3.593 (3)	172
C5—H5···O2W^vi^	0.95	2.50	3.429 (8)	166
C7—H7···Cl1^vii^	0.95	2.82	3.765 (2)	175

Symmetry codes: (iii) −*x*+1, −*y*, −*z*+1; (iv) −*x*, *y*, −*z*+1; (v) −*x*+1/2, *y*−1/2, −*z*+1; (vi) −*x*+1/2, −*y*+1/2, −*z*+1; (vii) −*x*+3/2, *y*+1/2, −*z*.
